# Spectacles and Eyeglasses, Their Forms, Mounting and Proper Adjustment

**Published:** 1896-03

**Authors:** 


Spectacles and Eyeglasses, their Forms, Mounting and Proper Adjustment. By R. J. Phillips, M. D., Adjunct Professor of Diseases of the Eye, Philadelphia Polyclinic and College for Graduates in Medicine; Ophthalmic Surgeon to the Presbyterian Hospital in Philadelphia, etc. Duodecimo, pp. 106, with forty-nine illustrations. Second edition, revised. Price, $1.00. Philadelphia: P. Blakiston, Son & Co. 1895.

The title of this book explains exactly what it contains. In it will be found much practical and reliable information which every ophthalmologist and optician should acquire. The new systems of enumeration of prisms have been incorporated in this edition and several new tables and cuts have been added. A. A. H.

